# Supplementary material for: Contribution of Risk and Resilience Factors to Suicidality among Mental Health-Help-Seeking Adolescent Outpatients: A Cross-Sectional Study
Source: J Clin Med. 2023 Mar 2;12(5):1974. doi: 10.3390/jcm12051974 (PMC10004343; doi:10.3390/jcm12051974)
Supplement: Supplementary file 1 [file jcm-12-01974-s001.zip › jcm-2208454-supplementary.pdf]

Resilience Counters but Does Not Mitigate the Association Between Peer-Victimization and Suicidality: A Cross-Sectional Study Among Adolescents Referring to an Outpatient Psychiatric Clinic

Supplementary material

Table S1. Multivariate logistic regression model with ASQ $\geq$ 1 as dependent variable; co-varying for depressive and anxiety symptoms.

Factor	OR (95% CI)	p
Age [a]	1.52 (0.96, 2.68)	.16
Sex [b]	1.51 (0.46, 5.13)	.50
Negative life events [a]	0.90 (0.46, 1.68)	.76
Parents' income [a]	1.12 (0.63, 2.03)	.69
Parents layoff during COVID-19 [b]	0.60 (0.12, 2.45)	.49
Peer victimization [a]	3.67 (1.76, 8.72)	**.001**
Resilience factors score [a]	0.37 (0.14, 0.89)	**.033**
Resilience factors score * Peer victimization [a]	1.48 (0.87, 2.64)	.16

PHQ-8 (i.e., PHQ-9 scores excluding suicidal ideation item) and GAD-7 scores were introduced as covariates to the model

[a] Standardized-scores

[b] Binary-values
